# Sale of Professor Koch's Lymph

**Published:** 1891-05-09

**Authors:** Von Gossler

**Affiliations:** Berlin


### Sale of Professor Koch's Lymph.

The following is a translation of a notice in the German *Official Gazette* (Berlin) for March 13th: Professor Dr. Koch has published in the *German Medical Gazette* of the 15th January last a description of the manner in which the lymph, discovered by him for the cure of tuberculosis, is prepared, and according to this description it appears that this remedy comes within the provisions of §1 of the Imperial Ordinance of the 27th January of last year, and can therefore, with the exception of wholesale trade, only be sold by licensed chemists (apothecaries). Chemists can at present obtain the lymph, prepared under the personal supervision of the inventor, only through his authorised representative, Dr. Libbertz, 28, Lüneburgerstrasse, Berlin, N.W. It is supplied to them in special bottles of a capacity of one or five cubic centim. These bottles have glass stoppers covered with bladder and secured by a leaden seal bearing the letter "L." On one side they bear the name "Tuberculinum Kochii" in white letters on a black ground, on the other side a white label with the signature of Dr. Libbertz and a note giving the date on which the lymph was prepared. Each bottle is accompanied by a printed paper containing instructions for its use. With regard to the preservation and sale of the remedy in chemists' shops, the following regulations must be observed: 1. The "Tuberculinum Kochii" must be kept in the poison cupboard, and in the division reserved for alkaloids. 2. It can only be sold in the original bottles, and only upon the written order of an approved physician, and delivered to such physician himself, or to a person authorized by him. 3. A record of the purchase and sale of the remedy must be kept in a special book, in which each bottle must be entered. The contents of the bottle, the date of preparation, of purchase and of sale, the name of the physician to whom it has been sold, or eventually the removal of the unsold bottle from the shop, must all be noted. 4. If a bottle has not been sold within six months of the date of preparation marked upon it, it can no longer be sold or otherwise disposed of, but must be removed from the shop. Such bottles will be exchanged by Dr. Libbertz for others containing freshly prepared lymph, free of charge. 5. The price of the "Tuberculinum Kochii" is hereby fixed (exclusive of the cost of packing) at 6 marks for a bottle containing 1 cubic centim., and at 25 marks for one containing 5 cubic centim. I have the honour to request you to cause the above regulations to be communicated to the chemists in your district for their guidance, and also to take steps to insure their due observance.

(Signed) Dr. von Gossler.

Berlin, March 1st, 1891.

To the Governors of Provinces and the President of Police in Berlin.

